# Multiwell Raman plate reader for high-throughput biochemical screening

**DOI:** 10.1038/s41598-021-95139-8

**Published:** 2021-08-03

**Authors:** Hiroyuki Kawagoe, Jun Ando, Miwako Asanuma, Kosuke Dodo, Tetsuya Miyano, Hiroshi Ueda, Mikiko Sodeoka, Katsumasa Fujita

**Affiliations:** 1 Department of Applied Physics, Osaka University, 2-1 Yamadaoka, Suita, Osaka 565-0871 Japan; 2 RIKEN Cluster for Pioneering Research and RIKEN Center for Sustainable Resource Science, 2-1 Hirosawa, Wako, Saitama 351-0198 Japan; 3 Laboratory for Medicinal Chemistry Research, Shionogi and Co., Ltd., 3-1-1 Futaba-cho, Toyonaka, Osaka 561-0825 Japan; 4 Advanced Photonics and Biosensing Open Innovation Laboratory, AIST-Osaka University, 2-1 Yamadaoka, Suita, Osaka 565-0871 Japan; 5 Transdimensional Life Imaging Division, Institute for Open and Transdisciplinary Research Initiatives, Osaka University, 2-1 Yamadaoka, Suita, Osaka 565-0871 Japan

**Keywords:** Analytical chemistry, Applied optics

## Abstract

Although Raman spectroscopy has been used for the quantitative analysis of samples in many fields, including material science, biomedical, and pharmaceutical research, its low sensitivity hindered the application of the analytical capability for high-throughput screening. Here, we developed a high-throughput Raman screening system that can analyze hundreds of specimens in a multiwell plate simultaneously. Multiple high numerical aperture (NA) lenses are assembled under each well in the multiwell plate to detect Raman scattering simultaneously with high sensitivity. The Raman spectrum of 192 samples loaded on a standard 384-well plate can be analyzed simultaneously. With the developed system, the throughput of Raman measurement was significantly improved (about 100 times) compared to conventional Raman instruments based on a single-point measurement. By using the developed system, we demonstrated high-throughput Raman screening to investigate drug polymorphism and identify a small-molecule binding site in a protein. Furthermore, the same system was used to demonstrate high-speed chemical mapping of a centimeter-sized pork slice.

## Introduction

High-throughput screening (HTS) has been a key technique in the recent pharmaceutical and biological studies. In HTS, the combination of well plates and plate readers allows us to analyze tens to hundreds of samples rapidly and comprehensively. For example, in the pharmaceutical industry, HTS has been employed to effectively identify the lead compounds for new drug discoveries from massive compound libraries^1,2^. In chemical and biological research, HTS provides the opportunity to investigate enormous combinations of biochemical interactions between drug molecules and cells in a practical experimental period^3,4^. In the current HTS, optical methodologies have been widely adopted owing to the non-destructive and high-speed analytical capabilities. Especially, fluorescent and luminescent assays are typically used due to their variations of available reporters^2,3,5^.

Raman spectroscopy can be a powerful screening tool because the Raman spectrum reflects the molecular vibration useful for investigating a sample with the information of molecular structures, molecular species, crystallinity, protein conformation, and so on^6–10^. Since Raman scattering is a general optical effect seen in almost all kinds of molecules, it does not require sample preparation, unlike fluorescence assays that may alter sample conditions and reactions in biological systems. In addition, Raman spectroscopy can be applied to samples in any phase (solid, liquid, and gas), allowing a wide range of applications, including live cell analysis and drug analysis, as seen in successful demonstrations in previous reports^8–10^.

To fully utilize the powerful capability of the sample analysis of Raman spectroscopy in screening applications, it is inevitable to improve the throughput of spectrum detection. The throughput of conventional Raman screening systems has been restricted by the observation area that can be measured at a single exposure. Since Raman scattering is a weak optical phenomenon, it is desirable to use a high numerical aperture (NA) lens to collect Raman scattering photons as much as possible. However, high NA lenses can observe only a small area, which is inversely proportional to the NA of the lens. For example, when using an objective lens with an NA of approximately 1.0 (the corresponding magnification is typically 20–100), the observation area is limited to less than 1 mm^2^. The limited observation area requires repetitive Raman measurements for the number of analytes, resulting in a considerably long screening time. In fact, the previous Raman screening conducted by Raman microscopes required a long measurement time of more than several tens of minutes to hours for tens of samples^11–14^. Raman imaging instruments employing a low-NA lens have been developed to observe a large observation area^15^. However, the low detection efficiency with the low NA lens and the requirement of multiple measurements at different detection bands for spectral analysis have limited the throughput of the measurement. Therefore, it has been difficult to achieve high-throughput Raman screening practically.

In this research, we developed a multiwell Raman plate reader that enables the simultaneous detection of Raman spectra from multiple analytes placed in a standard well plate. To demonstrate the utility of the Raman plate reader for high-throughput screenings, we conducted experiments using different types of samples. As one of the typical applications of Raman spectroscopy in the pharmaceutical industry, we performed the detection of drug polymorphs and successfully demonstrated the recognition of crystal forms after recrystallization. We also demonstrated the high-throughput Raman screening using surface-enhanced Raman scattering (SERS) spectroscopy^16,17^. The combination of SERS and the multiwell Raman plate reader can further improve the throughput of Raman screening. As a demonstration of SERS screening, we have improved the throughput of alkyne-tag Raman screening (ATRaS), an effective technique for finding small-molecule binding sites in proteins^18^. This experiment also confirmed the feasibility of high-throughput SERS screening with complex samples consisting of aggregations of metal nanoparticles and small molecules. Other than the applications using a multiwell plate, the optical design of the Raman plate reader allows us to apply Raman spectroscopy for the analysis of relatively large samples. Especially in the food industry, there is a demand to check the quality, chemical composition, and contamination of foods of various sizes. We have demonstrated the measurement of Raman spectra from multiple spots in a slice of pork meat to verify the capability of food analysis using the Raman plate reader.

## Results

### Multiwell Raman plate reader for high-throughput Raman screening

**Figure 1:**
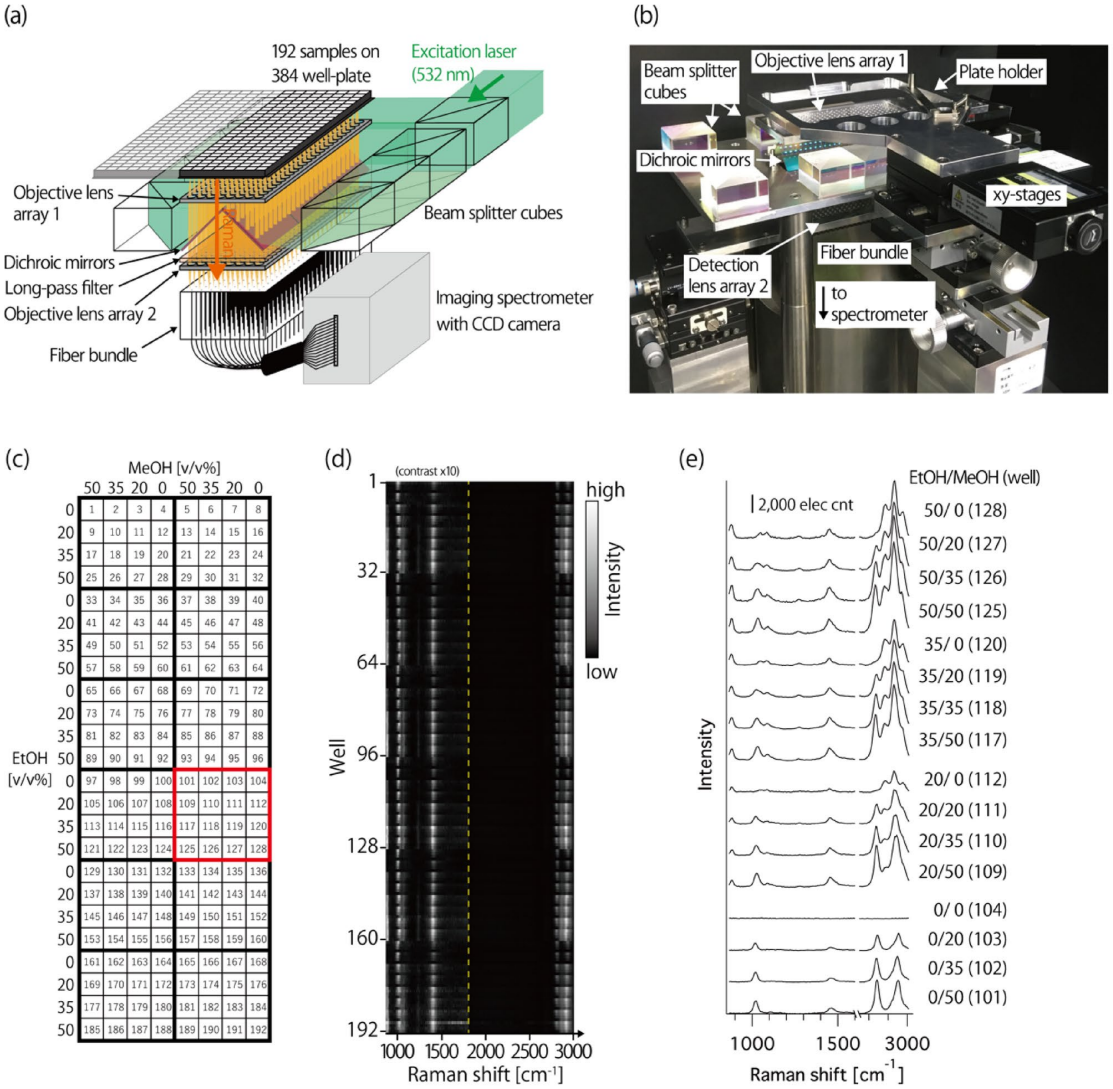
Multiwell Raman plate reader for high-throughput Raman screening. (**a**) Schematic and (**b**) photograph of the developed Raman plate reader. (**c**–**e**) Proof of concept for the simultaneous Raman measurement of 192 analytes. (**c**) Sample preparation of 192 mixtures loaded on a 384 well plate with different mixing ratios of ethanol and methanol. (**d**) Raman spectra of the 192 mixtures observed in 20 s. For clear illustration, the image contrast in the fingerprint region (865–1800 cm^−1^) is enhanced 10 × compared to that in silent- and CH-region (> 1800 cm^−1^). (**e**) Raman spectra observed in neighboring wells surrounded by a red square in (**c**).

The developed Raman plate reader employed multiple objective lenses, where high-NA lenses face each well of a well plate to collect the Raman scattering generated from each well simultaneously, realizing the effective signal collection with a high NA regardless of the number of analytes (Fig. 1a,b). We constructed objective lens arrays in which 192 small, semispherical lenses with NAs of 0.51 were arranged into 8 × 24 matrices with a separation of 4.5 mm between the lens centers, which matches the arrangement of 192 wells in half of the standard 384 well plate. This allows the simultaneous Raman measurement of 192 samples on a well plate with high efficiency in signal collection. Raman scattering photons corrected by the high-NA objective lens arrays were delivered to an imaging spectrometer using 192 optical fibers, and 192 Raman spectra were observed simultaneously with a two-dimensional CCD camera. For the simultaneous Raman excitation of 192 samples, large-area illumination optics composed of beam splitter cubes and dichroic mirrors were also developed. The plate holder and objective lens array were placed on an xy- and z-stage, respectively, so the focus position in a well could be moved during Raman measurement for detection with area averaging. Raman imaging at the 192 measurement positions can be also achieved by moving the sample plate sequentially. The achievable spatial resolution is ~ 1.8 µm, which is defined by the NA of the fiber. Complete information about the optical configuration is in “Methods” section.

For quantitative spectral analysis and comparison, numeric post-processing was applied to calibrate the differences in detection efficiency and spectral axis among 192 detection channels (well positions), mainly caused by optical aberrations occurring in the imaging spectrometer. For the calibration, the Raman spectrum of ethanol solution was measured at all of the 192 wells as a reference before the sample measurement. To compensate for distinctions in the detection efficiency in each well, the detected signals were multiplied by the channel-dependent calibration factors obtained from the Raman intensity of the reference (at 2930 cm^−1^). The spectral axis was calibrated independently using Raman peaks of ethanol (884, 1454, and 2930 cm^−1^) at each well.

As a proof of concept for the high-throughput Raman measurement of multiple analytes, we measured the mixtures of ethanol, methanol, and water with the developed Raman plate reader. The mixtures at different mixing ratios were loaded into 192 wells on a 384 well plate (Fig. 1c). The excitation laser power was set to ~ 7.5 mW/well. The exposure time and the CCD read-out time were 20 s and 268 ms, respectively. Figure 1d shows the resultant Raman spectra of the 192 mixtures taken by a single exposure. We confirmed that 192 Raman spectra were measured separately on a single CCD camera at the same time. Figure 1e plots the Raman spectra measured at neighboring wells indicated by a red square in Fig. 1c. In each Raman spectrum, the intensity of Raman peaks of ethanol (at 884; 1052; 1096; 1276; 1454; 2880; 2930; and 2974 cm^−1^)^19^ and methanol (at 1037; 1453; 2840; and 2949 cm^−1^)^20^ are in good agreement with the mixing ratios of ethanol and methanol at each well. Furthermore, no crosstalk between detection channels (wells) was observed in the spectra. In this measurement, the throughput of Raman screening was 20 s for 192 samples. This result clearly indicates that the developed Raman plate reader allows the high-throughput, quantitative-Raman analysis of multiple analytes.

### High-throughput Raman screening of drug polymorphism

By using the developed Raman plate reader, we performed high-throughput Raman screening to investigate the polymorphs of drug crystals. Drug polymorphism is the ability of a substance to crystallize into more than two forms. Investigation of drug polymorphism is essential for quality control on drug development because the important physicochemical properties of drugs, such as stability, solubility, and powder characteristics drastically change depending on its crystalline form^21,22^. Previous studies showed that Raman spectroscopy is a promising technique for investigating drug polymorphism owing to its high discrimination capability and minimal sample preparation requirements^11,12,14,23^. However, the throughput of Raman screening has been limited by a single-point measurement scheme based on a Raman microscope.

**Figure 2:**
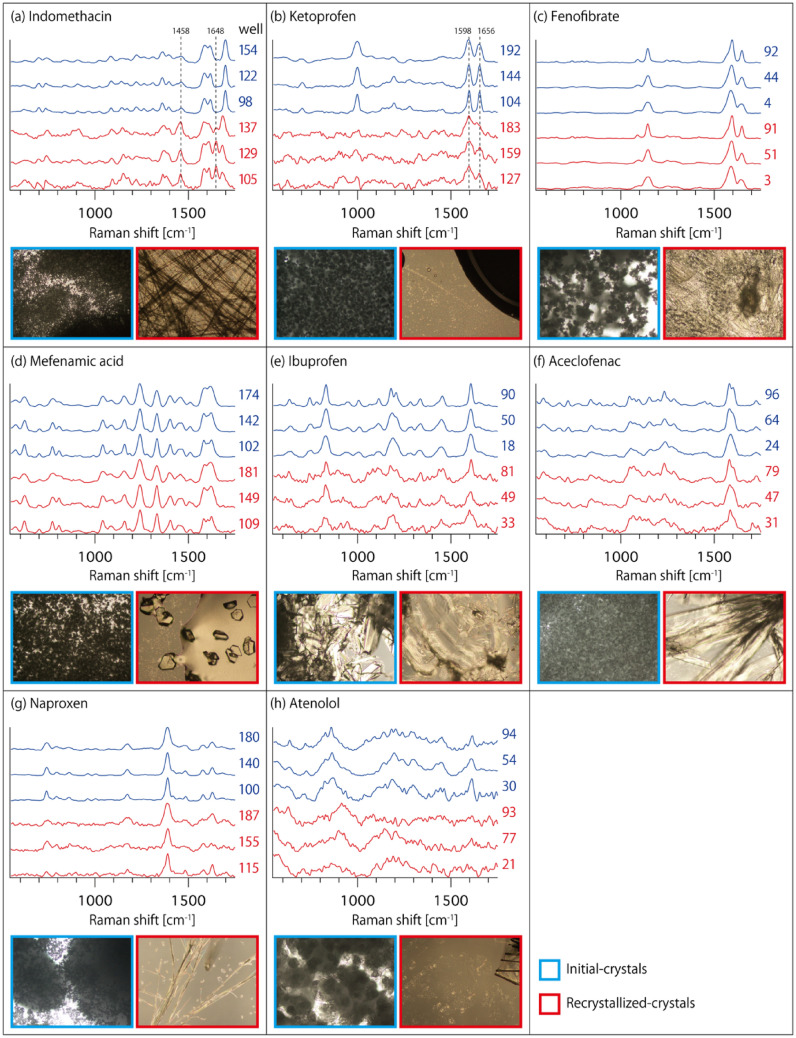
Representative Raman spectrum of drug crystals in wells for the investigation of drug polymorphism. (**a**) Indomethacin, (**b**) ketoprofen, (**c**) fenofibrate, (**d**) mefenamic acid, (**e**) ibuprofen, (**f**) aceclofenac, (**g**) naproxen, and (**h**) atenolol. Raman spectra of initial and recrystallized crystals are shown in blue and red, respectively. Each spectrum is normalized in intensity. Representative microscopic images of crystals are also shown for each drug molecule. The image size is 1.4 × 1.0 mm^2^.

For eight drug molecules, initial and recrystallized crystals from methanol were prepared in 192 wells of a 384 well plate (Fig. S1), and Raman spectra were observed using the developed Raman spectrometer. Figure 2 shows the representative Raman spectra of initial and recrystallized crystals for the eight drug molecules measured in 245 s. All Raman spectra at the 192 wells are shown in Fig. S2. Indomethacin and ketoprofen (Fig. 2a,b) show different Raman spectra before and after recrystallization, indicating the transformation of the drug crystal structure. In indomethacin, Raman peaks were observed at 1584; 1618; and 1698 cm^−1^ for initial crystals, whereas the new Raman peaks appeared at 1458 and 1648 cm^−1^ after recrystallization, which are consistent with Raman peaks of indomethacin in γ-form and α-form, respectively^12,24–26^. In ketoprofen, the peak intensity ratio of 1656 cm^−1^ against 1598 cm^−1^ was decreased after recrystallization, which arose from an amorphization of ketoprofen, as previously reported^27–29^. The assignment of the Raman peaks of indomethacin and ketoprofen is listed in Table 1. Conversely, no significant changes in Raman spectra between initial and recrystallized crystals were observed for the other six drug molecules (Fig. 2c–h). As shown in supplementary information (Fig. S2), part of the wells did not show a Raman spectrum with a signal-to-noise ratio (SNR) sufficiently to indicate the crystal form. This is because recrystallization occurred at the edge of the well, with only a few precipitates in the area of Raman measurement. We note that the throughput of the Raman screening of drug molecules with the developed Raman plate reader can be further improved if the number of recrystallized crystals can be increased with the optimal preparation method. In fact, for initial crystals, the Raman spectrum with high SNR was observed within 30 s exposure (Fig. S3).

**Table 1:** Assignment of Raman peaks of indomethacin and ketoprofen.

Peak wavenumber (cm^−1^)	Molecular vibration mode	References
**Indomethacin**		
Initial crystal (γ-form)		
1584	Ring C–C stretching	^30^
1618	C–O stretching of indole ring	^30^
1698	Benzoyl C=O stretching without H-bonding	^31^
Re-crystallized crystal (α-form)		
1458	In-plane indole ring deformation	^30^
1648	Benzoyl C=O stretching down-shifted due to H-bonding	^31^
**Ketoprofen**		
1656	Benzoyl C=O stretching	^29^
1598	C–C stretching	^29^

### High-throughput alkyne-tag Raman screening (ATRaS)

Alkyne-tag Raman screening (ATRaS)^18^ provides a highly efficient way to identify a small-molecule binding site in a protein, which is an emerging research area for better understanding of drug-protein interactions and biological functions of proteins^32–34^. In our previous paper^18^, small molecules were tagged with alkyne^6,35–38^ and detected by SERS to find small-molecule-modified peptides for successive mass spectrometry (MS). We successfully revealed the inhibitor-binding site in cathepsin B. However, the throughput of Raman spectroscopy for detecting alkyne signals was limited to few seconds per analyte due to the low Raman scattering cross-section.

**Figure 3:**
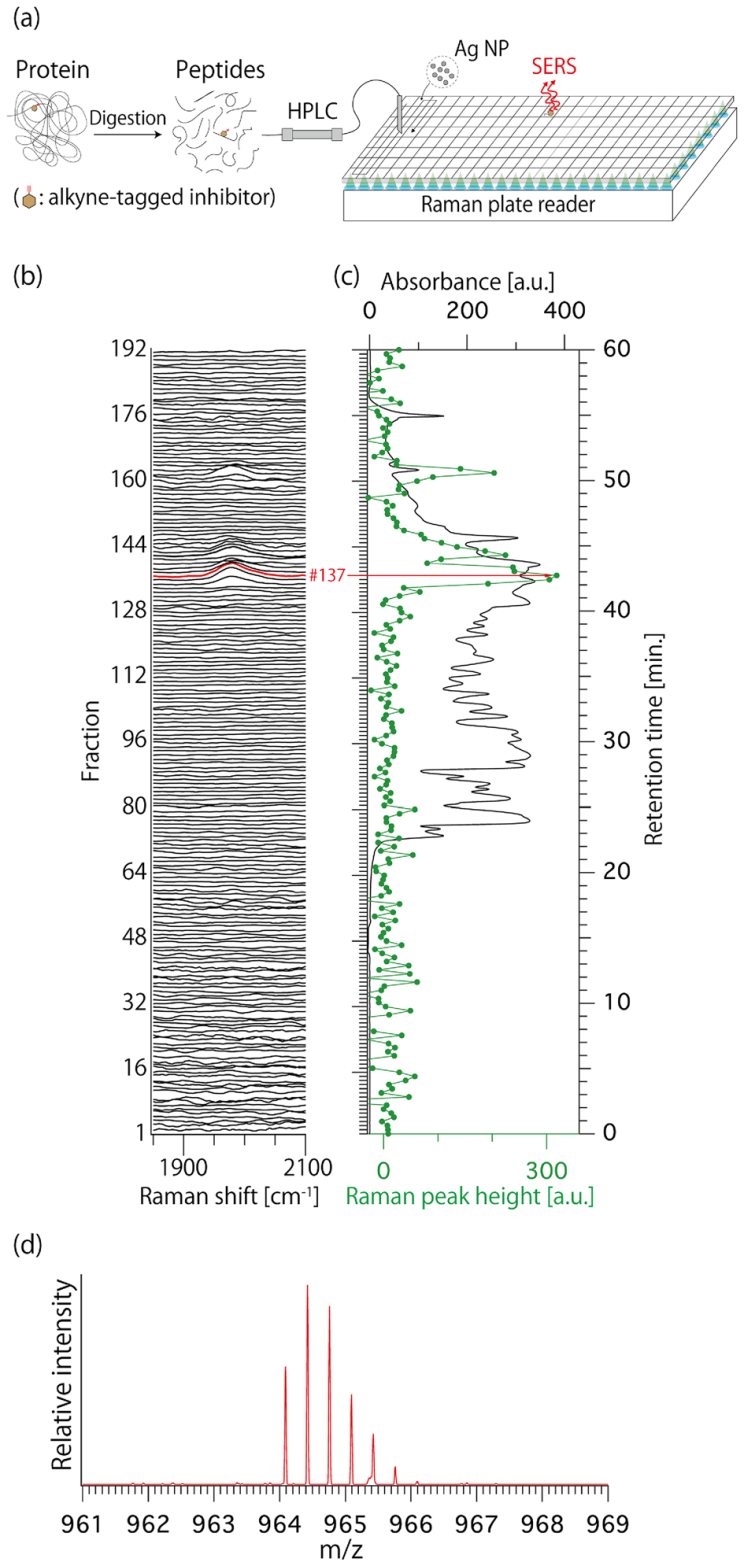
High-throughput ATRaS for the identification of the inhibitor binding site in a protein. (**a**) Schematic of SERS-ATRaS with the developed Raman plate reader. (**b**) Raman spectra of 192 fractions observed at 3 s. (**c**) Raman peak height at 1980 cm^−1^ (green) and UV absorbance at 215 nm (black) for each fraction. (**d**) Mass spectrum observed at fraction #137. The MS spectrum is good agreement with that of alt-AOMK labeled peptide, containing the amino-acid sequence of ^19^EIRDQGSCGSCWAFGAVEAISDR^41^, as reported in Ref.^18^.

We applied the multiwell Raman plate reader to improve the throughput of ATRaS. We prepared the sample in the same manner as our previous paper^18^, and briefly describe it here. The peptides digested from cathepsin B (CatB) after treatment with alkyne-tagged inhibitor molecules (alt-AOMK)^18^ were fractioned into 192 wells by using HPLC and a fraction collector; see Fig. 3a. After fractionation, a solution including silver nanoparticles was added to the 192 wells to increase the Raman signal by SERS.

Figure 3b shows the Raman spectra of the 192 fractions measured by the multiwell Raman plate reader with the measurement time of 3 s. The intensity of SERS signal of alkyne at 1980 cm^−1^ and the UV absorbance measured during HPLC fractionation are plotted with the fraction order in Fig. 3c. Similar to our previous results^18^, the strong SERS signals were also observed, which indicates that the SERS screening of peptides labeled with alt-AOMK was achieved rapidly and appropriately with the developed Raman plate reader. Since conditions were slightly different between the two experiments, we examined the fraction #137 by mass spectrometric analysis as the example of the strong Raman signal of alkyne (see “Methods”). As expected, this measurement identified an alt-AOMK-modified peptide (Fig. 3d) that is identical to the one obtained in our previous report^18^.

### High-speed chemical mapping of large-scale samples

**Figure 4:**
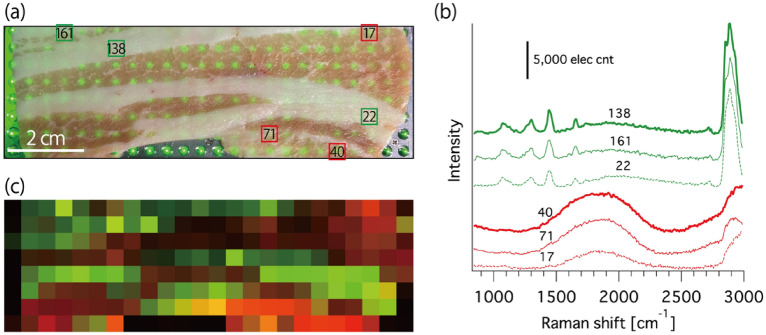
Chemical mapping of a large-scale sample measured by the developed Raman plate reader. (**a**) Photograph of a pork slice under illumination through multiple objective lenses. (**b**) Representative spectra obtained from the sample for the measurement time of 85 s. (**c**) Reconstructed chemical map of the sample. Green: fat (Raman intensity at 2890 cm^−1^), red: Zn-protoporphyrin IX (average intensity of fluorescence peak between 578 and 615 nm; 1496–2530 cm^−1^).

The multiwell Raman plate reader is also useful for high-speed and large-area chemical mapping, which has been anticipated in food analysis and studies of artwork^15,39–41^. By using our Raman plate reader, we can obtain spectroscopic information at hundreds of points spread in a large field of view (108 × 36 mm^2^) simultaneously. To demonstrate high-speed, large-area chemical mapping, we measured a pork slice; see Fig. 4a. The representative spectra observed from the sample in 85 s are shown in Fig. 4b. The spectra are categorized into two groups. The first is the Raman spectrum of lipid (1075; 1443; 2855; and 2885 cm^−1^) and protein [~ 1300 cm^−1^ (amide III) and ~ 1650 cm^−1^ (amide I)] in fat^40–42^, whereas the other is the fluorescence spectrum with a broad peak around 590 nm (~ 1850 cm^−1^) from Zn-protoporphyrin IX, which is the origin of the red color of meat^43,44^. Figure 4c maps the chemical information of the sample constructed from the observed spectra, which shows good agreement with a sample photo (see Fig. 4a). This result indicates that the developed spectrometer has the sufficient capability for obtaining a chemical map of a large sample at a high speed.

## Discussion

In this paper, we achieved high-throughput Raman screening of multiple specimens with a multiwell Raman plate reader that realizes the large-scale parallelization of Raman spectroscopic measurements for a multiwell plate platform. The multiwell Raman plate reader employing multiple high-NA lenses allows us to measure Raman spectra of 192 analytes on a 384 well plate simultaneously with high sensitivity. By using the multiwell Raman plate reader we developed, we demonstrated the identification of drug polymorphs and small-molecule-modified-peptides in a protein. These demonstrations proved the effectiveness of the developed multiwell Raman plate reader in high-throughput screening in pharmaceutical and biochemical fields by providing rich information based on molecular vibration. In addition, as a proof of concept for the high-speed chemical mapping of large-scale samples, the high-speed Raman chemical mapping of a centimeter-size biological tissue was shown.

We can estimate the throughput enhancement by the multi-well Raman plate reader as follows. We assume that the excitation wavelength and excitation power at a focus spot on the sample are the same for the developed system and a conventional Raman system with a single focus spot. The amount of Raman scattering photon detected by a detector per unit time depends on the NA of the objective lens, which is typically 0.9 for the conventional Raman system with a dry objective lens and 0.51 for the developed plate reader. Considering a solid angle for light detection, the detection efficiency with the developed system is about one fourth of the conventional Raman system. However, the developed system can measure 192 Raman spectra at the same time, which increases the throughput by 48 times when detecting multiple analytes. Furthermore, for thick samples such as solutions in wells and biological tissues, the amount of detected photon is accumulated with depth at a rate of 1/NA^2^. As a result, the detection efficiency of the two modalities becomes almost the same, and the throughput can be increased by two orders of magnitude by parallel detection. In fact, in conventional Raman systems, the throughput is reduced by mechanical scanning to move the observation area.

Here, we discuss future developments to enhance the analytical capability of the Raman plate reader. The number of samples that can be observed simultaneously and the spectral resolution can be further improved by optimizing the imaging spectrometer. In our experiments, the spectral resolution of the Raman spectra obtained from the top and bottom of the fiber bundle was lower than that of the central part due to the optical aberrations and vignetting in the spectrometer. The total height of the bundle at the entrance of the spectrophotometer was 24 mm, but the spectral quality at the imaging spectrometer could only be maintained within an image height of 20 mm. This also limits the number of wells that can be observed simultaneously with the current Raman plate reader. This limitation can be solved by developing an imaging spectrometer capable of measuring larger objects without aberrations and vignetting. Spectral deconvolution algorithms would be also helpful in retrieving spectral resolving power^45,46^.

In future applications of high-throughput Raman screening, cell-based biological studies are promising. The various investigations related to drug responses and biological activities using cells have been successfully demonstrated by Raman spectroscopy^47–49^. The developed Raman plate reader can drastically improve the throughput of the Raman measurement during an experimental period, which allows us to investigate many combinations of drugs and environmental conditions efficiently. The capability of the simultaneous detection of spectroscopic information at hundreds of points in a sample will also expand the application of Raman spectroscopy in rapid and large-area inspection, such as intraoperative rapid diagnosis, food analysis, and the rapid inspection of semiconductor products^41,50,51^.

## Methods

### Multiwell Raman plate reader

The Raman plate reader was designed to simultaneously observe the Raman spectrum of 192 analytes loaded in 192 wells on a standard 384-well plate. The plate reader consisted of a large-area illumination unit and a parallel detection unit; see Fig. 1a.

For Raman excitation, a continuous-wave (CW) laser at 532 nm (Verdi V-18, Coherent) was used. The laser beam was expanded by two lens pairs and introduced to the large-area illumination unit. In the illumination unit, the beam was first divided into two beams and then further divided into three beams for each divided beam by using non-polarized beam splitter cubes [36 × 36 × 22 mm in size (OptoSigma)]. The splitting ratios of each beam splitter cube were optimized to achieve the same laser power among the six divided beams. The divided beams were reflected by dichromic mirrors (DML 545 nm, Asahi Spectra), which were placed at 45° at the center of the system, to the sample plate placed at the top of the plate reader. On the sample plate, 192 focus spots were created through an objective lens array that consisted of 192 small semispherical lenses with NA of 0.51 (HSPL-4-2, Chuo Precision Industrial), which are arranged into 24 × 8 matrices with a separation of 4.5 mm between the lens centers to match the coordinate of 192 wells on a 384 well plate. To obtain the averaged Raman spectrum of dispersed samples in each well, the position of the focal spot in a well can be moved during laser exposure by using xy- and z-stages equipped for the plate holder and objective lens array, respectively.

Raman spectra generated from the 192 different analytes were collected simultaneously by using an optical fiber bundle. The fiber bundle consisted of 192 multi-mode fibers (50 μm core and 125 μm clad, and NA of 0.2), aligned to the same coordinate with the objective lens array. We duplicated the objective lens array for light coupling into the fibers. Long-pass filters (LWPF 540 nm, Asahi Spectra) were used to remove the excitation laser line. At the output end of the fiber bundle, the 192 fibers were aligned into one dimension (1D) along a slit of an imaging spectrometer (IsoPlane 320 Advanced, Princeton). Raman spectra of 192 analytes delivered by 192 fibers were simultaneously captured by a 2D-cooled charge-coupled device (CCD) camera (PIXIS 400 BeX, Princeton), which was placed so that the long axis of the sensor (26.8 mm tall) was parallel to the slit of the spectrometer. Using a blazed grating with 300 lines/mm, the detection band of the Raman spectrum was about 800–3000 cm^−1^ with a spectral interval of about 5.5 cm^−1^/pixel.

### Sample preparation and spectral analysis for Raman screening of drug polymorphs

Ibuprofen, atenolol, and aceclofenac were purchased from TOKYO CHEMICAL INDUSTRY CO., LTD. Fenofibrate, naproxen, and mefenamic acid were purchased from Sigma-Aldrich Japan. Indomethacin and ketoprofen were purchased from FUJIFILM Wako Pure Chemical Corporation.

As shown in Fig. S1, initial and recrystallized crystals of the eight drug molecules were added into 192 wells of a glass-bottom 384-well plate (EZ-View, IWAKI). For the initial crystals (rows B, D, F, and H in the plate), powder samples were added with a spatula. For the recrystallized crystals (rows A, C, E, and G), 100 µL of 0.5 mg/mL methanol solution of each drug was added to each well and evaporated overnight at room temperature.

In the Raman screening of drug crystals, we measured the Raman spectra of the drug crystals seven times with an exposure time of 30 s for each to integrate the signals without saturating the CCD camera. The total screening time was 245 s, including a CCD read-out time of 35 s. During laser exposures, the sample plate was continuously scanned to obtain the averaged Raman spectrum of crystals spread in each well. The laser power applied for each well was about ~ 7.5 mW. We used moving average filter and modified polynomial fitting^52^ to improve the SNR and remove the fluorescence background generated by drug crystals. The detection range of the Raman spectrum was 500–2200 cm^−1^ and a 300 lines/mm blazed grating was used.

### Sample preparation for well plate containing peptide fractions

The sample was prepared as in our previous report^18^ with some modifications. The preparation of enzymatic digests of Alt-AOMK-labeled cathepsin B was as below. Twenty μg of human liver cathepsin B (CatB; CALBIOCHEM) was dissolved in 500 μL of acetate buffer [50 mM sodium acetate (pH 5.6), 5 mM MgCl_2_, 2 mM dithiothreitol (DTT)], and the solution was kept for 15 min at room temperature. Alt-AOMK dissolved in DMSO was added to the solution at the final concentration of 30 μM, and the mixture was incubated for 1 h at 37 °C. After incubation, the proteins in the solution were purified by trichloroacetic acid (TCA) precipitation. The precipitate was dissolved in 15 μL of denaturing buffer [7 M guanidine hydrochloride (GuHCl), 0.5 M Tris–HCl (pH 8.5)], and the solution was incubated for 1 h at 37 °C. After the reduction and alkylation of proteins with 125 mM DTT and 250 mM iodoacetamide, respectively, Milli Q water was added to the solution to reduce the concentration of GuHCl to less than 0.7 M, and *n*-decyl-β-d-glucopyranoside (DG) was added to the solution at the final concentration of 0.01% (w/v). For the enzymatic digestion of proteins, 1 μg of lysyl endopeptidase (Mass Spectrometry Grade; FUJIFILM Wako Chemical) was added to the solution. After incubating the solution for 2 h at 37 °C, 1 μg of Sequencing Grade Modified trypsin (Promega) was added to the solution and incubated overnight at 37 °C.

The well plate containing peptides was prepared as follows. Enzymatic digests of alt-AOMK-labeled CatB (equivalent to 6 μg protein) were fractionated on an UltiMate 3000 RSLC, nano LC system (Themo Fisher Scientific) equipped with both an MU701 UV detector (GL Science) and a microfraction collector Probot (Thermo Fisher Scientific). Peptides were trapped on an Acclaim PepMap100 C18 trap column (0.3 × 5 mm, 5 µm, Thermo Fisher Scientific) at a flow rate of 30 μL/min and separated on an analytical column (Acclaim PepMap100 C18 NanoViper, 0.075 × 150 mm, 2 μm, Thermo Fisher Scientific) at a flow rate of 250 nL/min. For the HPLC solvent, mobile-phase A [distilled water containing 0.1% [v/v] trifluoroacetic acid (TFA)] and mobile-phase B [0.1% [v/v] TFA, and 90% [v/v] acetonitrile (MeCN)] were used. Gradient conditions using mobile phase A and mobile phase B were as follows: 4% B (10 min), 4–39% B (25 min), 39–78% B (10 min), 78–99% B (0.1 min), 99% B (3.9 min), 99–4% B (1 min), and 4% B (10 min). The UV absorbance was monitored at 215 nm. The eluate was fractionated into a processed glass-bottomed 384-well plate preloaded with 30 μL of 0.3% (v/v) TFA solution containing 0.001% (w/v) DG in each well at 20 s intervals. After the fractionation of peptides into the plate, 5 μL of the sample was transferred to a new 384 polypropylene plate for mass spectrometric analysis.

### Experimental procedure and data analysis for high-throughput ATRaS

Before the Raman measurement of the well plate containing fractioned peptides, we added the solution including silver nanoparticles with a diameter of 40 nm into the plate and kept the plate overnight at 4 °C to accumulate aggregations of silver nanoparticles and peptides. SERS spectra of the peptides loaded into the 192 wells were observed with an excitation power of ~ 7.5 mW/well and an exposure time of 3 s (the readout time was 268 ms). In data analysis, the moving average filter and polynomial fitting^52^ were applied to improve SNR and remove background signals from the spectrum.

### NanoLC–MS and peptide identification

LC–MS/MS analysis was performed using an LTQ Orbitrap XL mass spectrometer (Thermo Fisher Scientific) equipped with a nanoESI source (Nikkyo Technos) and an UltiMate 3000 nano LC system (Thermo Fisher Scientific). Peptides were separated with an Acclaim PepMap 100 C18 analytical column NanoViper (0.075 mm × 150 mm, 3 µm, Thermo Fisher Scientific) after trapping on an Acclaim PepMap 100 C18 trap column NanoViper (0.1 mm × 20 mm, 5 μm, Thermo Fisher Scientific). For the analytical column, mobile-phase A (distilled water containing 0.1% [v/v] formic acid (FA) and 4% [v/v] MeCN) and mobile-phase B (MeCN containing 0.1% [v/v] FA) were used. Mobile-phase C (distilled water containing 0.1% [v/v] TFA) and mobile-phase D (MeCN containing 0.1% [v/v] TFA) were used for the trap column. The gradient condition of mobile phase A and mobile phase B was maintained at a flow rate of 250 nL/min. Survey scans were performed in the Orbitrap mass analyzer (resolution 60,000), and MS/MS spectra were acquired in the linear ion trap with data-dependent acquisition in the positive ion mode. The MS and MS/MS data were searched using MASCOT software (Matrix Science) via Proteome Discoverer (Thermo Fisher Scientific). The representative parameters were as follows: The precursor mass tolerance was 10 ppm, the fragment mass tolerance was 0.8 Da, the enzyme was trypsin, the maximum number of missed cleavage sites was three, and dynamic modifications were the carbamidomethylation of cysteine (+ 57.021464 Da), with the Alt-AOMK modification of cysteine (+ 376.142307 Da).

### Experimental procedure for high-speed chemical mapping of pork slice

A pork slice purchased from a market was placed on a quartz substrate (t = 0.17 mm) and measured by the Raman plate reader. The size of the pork slice was 100 × 40 mm^2^. For Raman measurement, the measurement time was 85 s, including 5 s readout time of the CCD camera. The excitation power was approximately 7.5 mW at each focus.

## Supplementary Information

Supplementary Figures.
