# Supplementary material for: Multiwell Raman plate reader for high-throughput biochemical screening
Source: Sci Rep. 2021 Aug 3;11:15742. doi: 10.1038/s41598-021-95139-8 (PMC8333358; doi:10.1038/s41598-021-95139-8)
Supplement: Supplementary file 1 — Supplementary Figures. [file 41598_2021_95139_MOESM1_ESM.pdf]

Supporting information for *Multiwell Raman plate reader for high-throughput biochemical screening*

Fig. S1: Sample preparation of drug crystals at 192 wells on a 384-well plate

Fig. S2: Raman spectra of drug crystals at 192 wells observed at 245 s. Raman spectra of initial- and recrystallized-crystals are shown in blue and red, respectively. Raman spectra with low SNR due to small numbers of precipitates in the Raman measurement area are shown in brown. For all Raman spectra, intensities are normalized. Representative microscopic images of crystals are also shown for each drug molecule. The image size is 1.4 x 1.0 mm^2 for initial and recrystallized crystals and 0.42 x 0.33 mm^2 for recrystallized crystals in small amounts.

Fig. S3: Raman spectra of initial crystals with a measurement time of 30 s.

	1	2	3	4	5	6	7	8	9	10	11	12	13	14	15	16	17	18	19	20	21	22	23	24
A	1	9	17	25	33	41	49	57	65	73	81	89	97	105	113	121	129	137	145	153	161	169	177	185
B	2	10	18	26	34	42	50	58	66	74	82	90	98	106	114	122	130	138	146	154	162	170	178	186
C	3	11	19	27	35	43	51	59	67	75	83	91	99	107	115	123	131	139	147	155	163	171	179	187
D	4	12	20	28	36	44	52	60	68	76	84	92	100	108	116	124	132	140	148	156	164	172	180	188
E	5	13	21	29	37	45	53	61	69	77	85	93	101	109	117	125	133	141	149	157	165	173	181	189
F	6	14	22	30	38	46	54	62	70	78	86	94	102	110	118	126	134	142	150	158	166	174	182	190
G	7	15	23	31	39	47	55	63	71	79	87	95	103	111	119	127	135	143	151	159	167	175	183	191
H	8	16	24	32	40	48	56	64	72	80	88	96	104	112	120	128	136	144	152	160	168	176	184	192

A, B 1-12: Ibuprofen
C, D 1-12: Fenofibrate
E, F 1-12: Atenolol
G, H 1-12: Aceclofenac
A, B 13-24: Indomethacin
C, D 13-24: Naproxen
E, F 13-24: Mefenamic Acid
G, H 13-24: Ketoprofen

At rows A, C, E, and G (shown in red), recrystallized crystals were prepared.

Fig. S1.

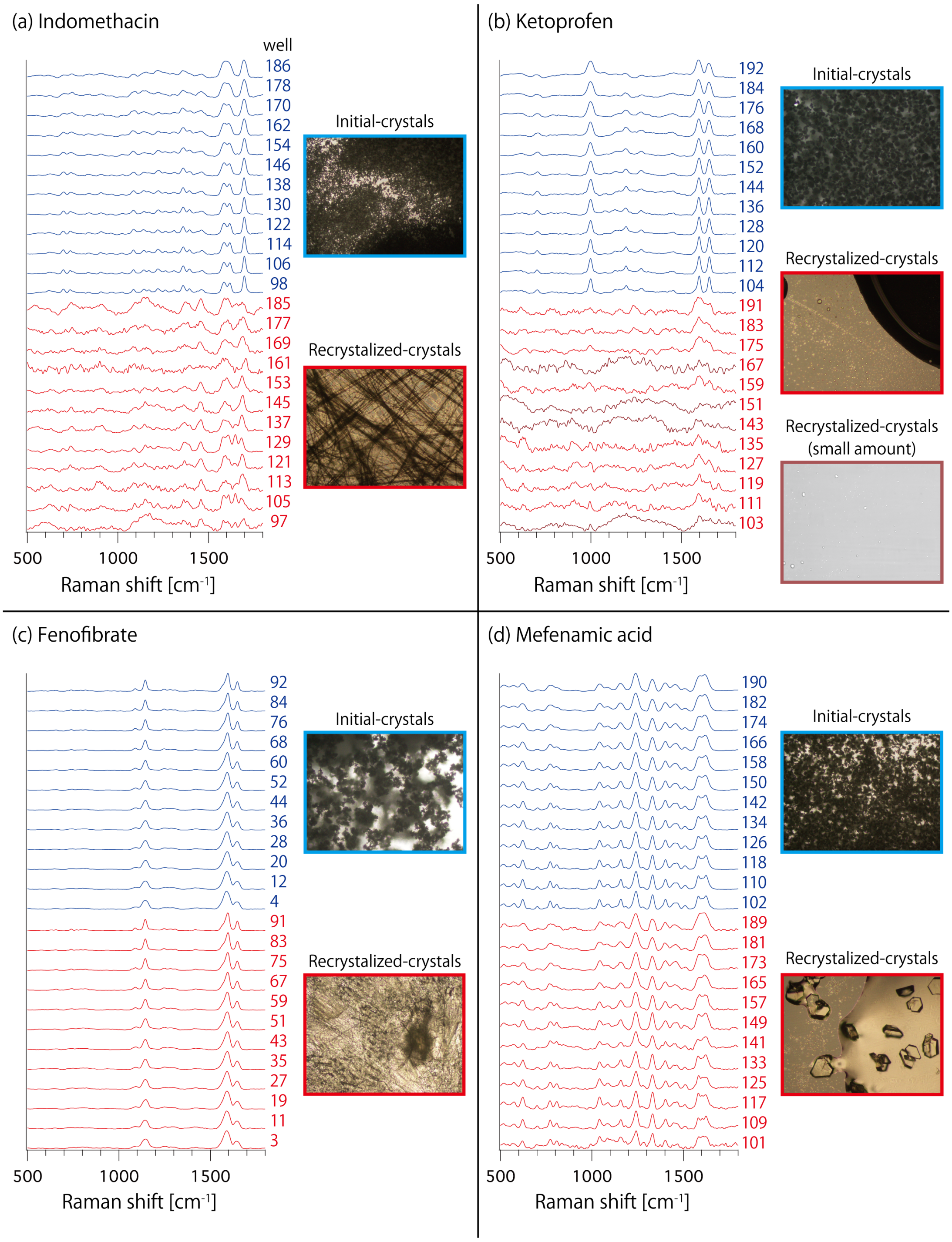

Fig. S2 (continue to the next page)

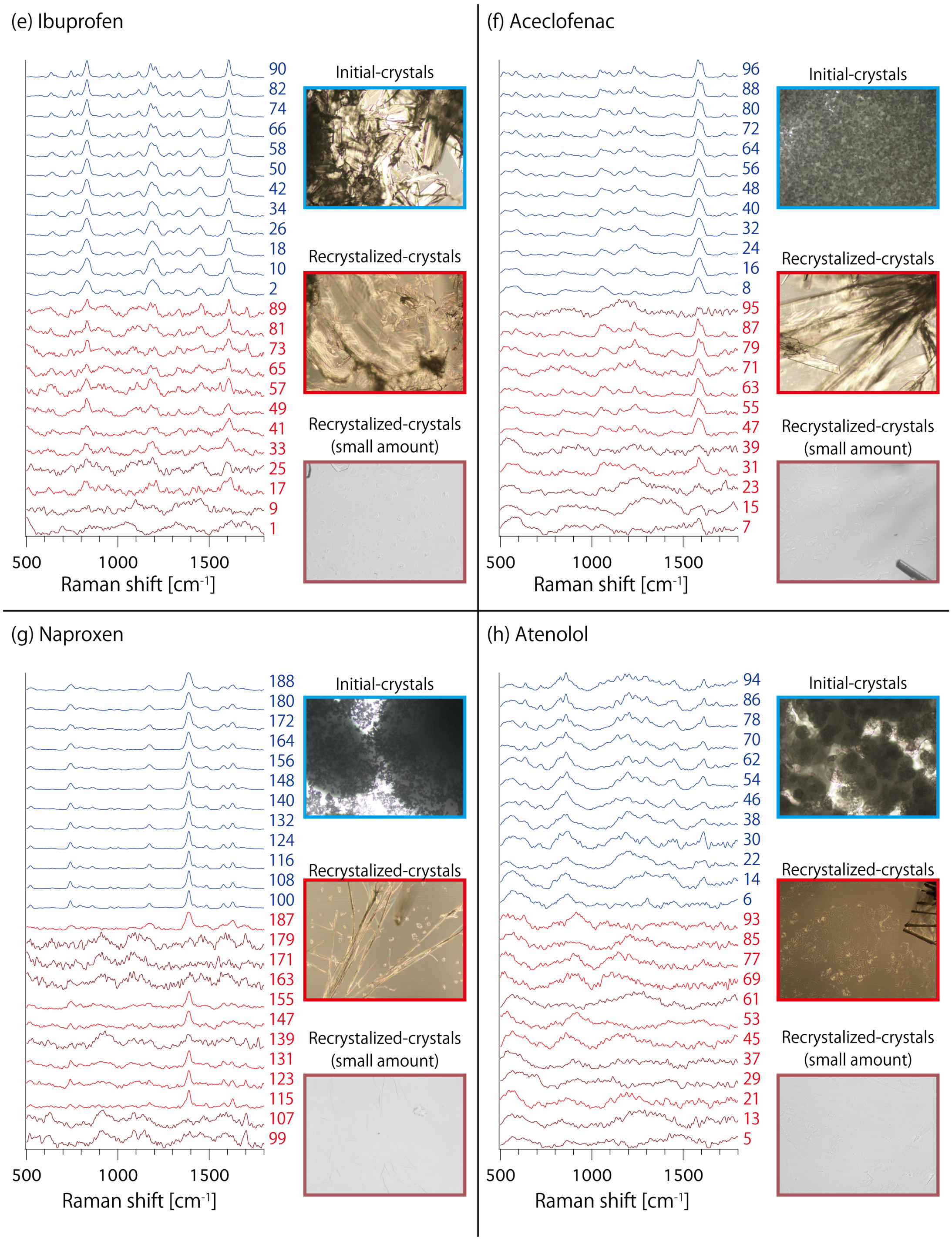
(e) Ibuprofen
90 82 74 66 58 50 42 34 26 18 10 2
89 81 73 65 57 49 41 33 25 17 9 1
500 1000 1500
Raman shift [cm-1]
Initial-crystals
Recrystalized-crystals
Recrystalized-crystals (small amount)
(f) Aceclofenac
96 88 80 72 64 56 48 40 32 24 16 8
95 87 79 71 63 55 47 39 31 23 15 7
500 1000 1500
Raman shift [cm-1]
Initial-crystals
Recrystalized-crystals
Recrystalized-crystals (small amount)
(g) Naproxen
188 180 172 164 156 148 140 132 124 116 108 100
187 179 171 163 155 147 139 131 123 115 107 99
500 1000 1500
Raman shift [cm-1]
Initial-crystals
Recrystalized-crystals
Recrystalized-crystals (small amount)
(h) Atenolol
94 86 78 70 62 54 46 38 30 22 14 6
93 85 77 69 61 53 45 37 29 21 13 5
500 1000 1500
Raman shift [cm-1]
Initial-crystals
Recrystalized-crystals
Recrystalized-crystals (small amount)

Fig. S2

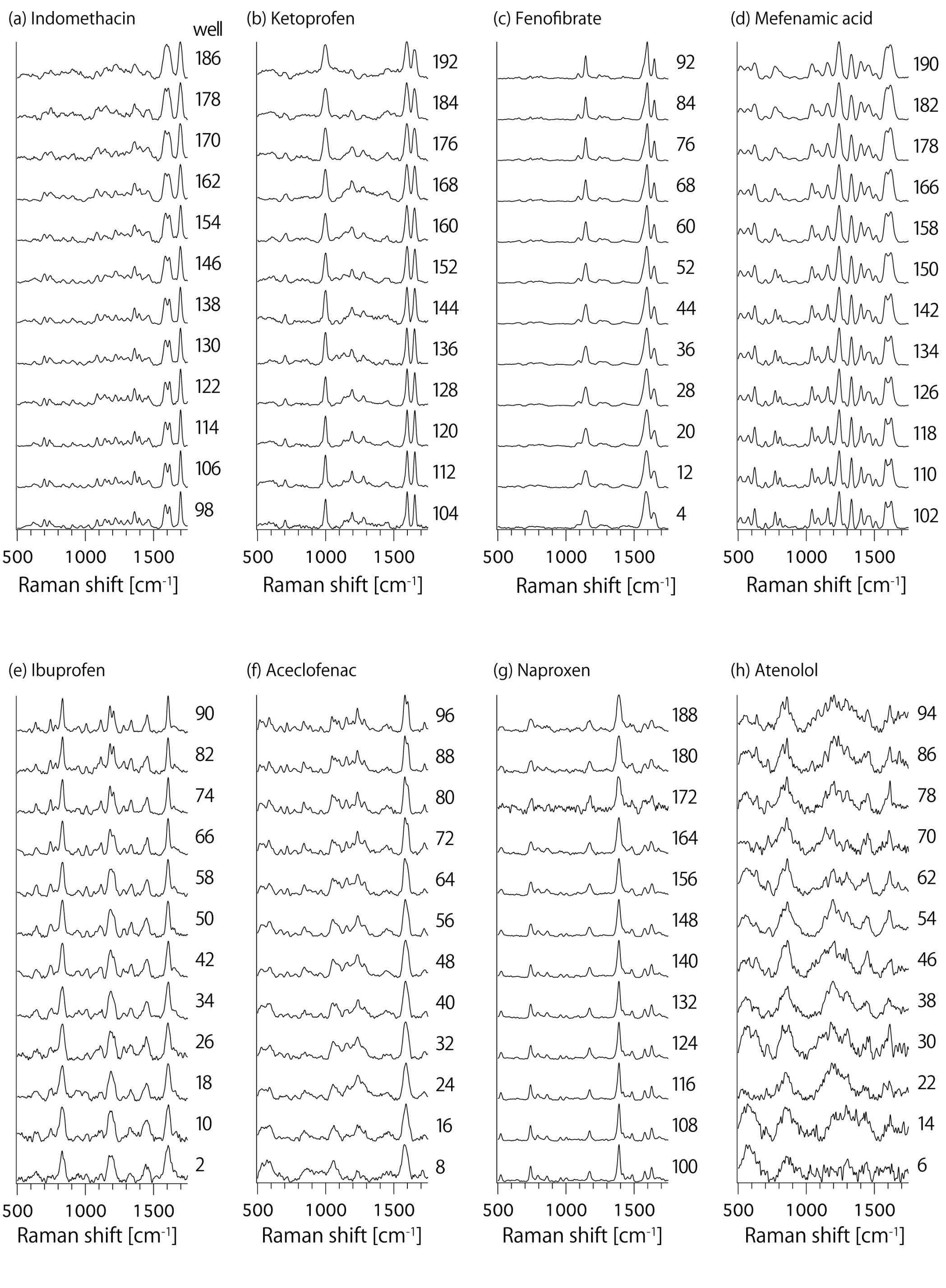
(a) Indomethacin
well
186
178
170
162
154
146
138
130
122
114
106
98
(b) Ketoprofen
192
184
176
168
160
152
144
136
128
120
112
104
(c) Fenofibrate
92
84
76
68
60
52
44
36
28
20
12
4
(d) Mefenamic acid
190
182
178
166
158
150
142
134
126
118
110
102
(e) Ibuprofen
90
82
74
66
58
50
42
34
26
18
10
2
(f) Aceclofenac
96
88
80
72
64
56
48
40
32
24
16
8
(g) Naproxen
188
180
172
164
156
148
140
132
124
116
108
100
(h) Atenolol
94
86
78
70
62
54
46
38
30
22
14
6
500
1000
1500
Raman shift [cm-1]

Fig. S3.
